# 1-Methyl-3-octylimidazolium Chloride—Sorption and Primary Biodegradation Analysis in Activated Sewage Sludge

**DOI:** 10.3390/molecules14114396

**Published:** 2009-11-02

**Authors:** Marta Markiewicz, Christian Jungnickel, Aleksandra Markowska, Urszula Szczepaniak, Monika Paszkiewicz, Jan Hupka

**Affiliations:** 1 Department of Chemical Technology, Chemical Faculty, Gdańsk University of Technology, ul. Narutowicza 11/12, 80-233 Gdańsk, Poland; 2 Faculty of Chemistry, University of Gdańsk, Sobieskiego 18, PL 80-233 Gdańsk, Poland

**Keywords:** ionic liquids, sewage sludge, biodegradation, imidazolium, sorption

## Abstract

Ionic liquids (ILs) are known to be non-volatile and thus to have low potential for atmospheric contamination or intoxication of humans by inhalation. However ILs have the potential to contaminate soil and water as they might be water soluble and can be sorbed onto solids. The investigation of possible natural ways of reducing the concentration of ILs in the environment is of high importance, especially because the requirement for biodegradable chemicals increases, together with pressure for reduction of incineration and landfill waste. It was found that the upper concentration threshold for primary biodegradation of 1-methyl-3-octylimidazolium chloride is 0.2 mM. At higher concentrations the dehydrogenase activity of the cells dropped markedly, indicating that the IL inhibits cell activity. This concentration is in good agreement with the minimal inhibitory concentration of the same compound found for a series of bacteria and fungi by this research group. The sorption of 1-methyl-3-octylimidazolium chloride was found to be significant, and the sorption coefficient was determined to be 98.2 L kg^-1^.

## Introduction

Ionic liquids (ILs) are a group of compounds usually composed of three exchangeable chemical moieties: a cationic head group, and head group’s organic substituent and an organic or inorganic anion. These three elements can be altered, thereby resulting in different physicochemical properties and thus allowing us to tailor the molecule’s features. Some ILs can be structurally analogous to surfactants and can therefore present surface activity [1]. The most significant advantages of ILs are commonly higher efficiency of some reactions, wide electrochemical potential window, better solvent properties, miscibility with water/organic solvents, recyclability and the most importantly from an environmental point of view – almost no measurable vapour pressure [2,3,4,5]. Therefore, ILs are unlikely to act as air contaminants or inhalation toxins. Nevertheless, they have a considerable potential to contaminate soil and water. The broad range of possible applications of ILs in industry forces intensive research into their possible distribution and behaviour in the environment. As a result of the focus on sustainable design, the environmental impact of ILs is often considered, including investigation of their biodegradation. Due to their inherent toxicity they also can significantly influence the performance of biological sewage sludge treatment systems. 

### The significance of biodegradability

A release of IL to the aquatic environments could potentially have catastrophic effects, due to the high solubility of some ILs in water, high thermal and chemical stability, potential toxicity and possible recalcitrance. The level of human toxicity has an obvious significance and direct impact on man and animals. Microbial toxicity, even though it may appear less obvious, has the ability to limit the level of biodegradation of these substances in the environment. Degradation of chemicals by microorganisms has a great potential due to broad, naturally occurring, microbial metabolic variety allowing for the transformation, degradation or even accumulation of an enormous range of compounds. One of the microbial communities most often used in biodegradation processes is activated sewage sludge. 

It should be mentioned that the possible biodegradation and biological activity of ILs may influence their environmental impact and distribution [6,7]. This will affect the environmental fate of the ILs and eventual bioremediation possibilities, for example the activity of sewage sludge, by adsorption on cellular and mineral surfaces [8,9]. Ionic liquids can adsorb on the surface of microorganisms and penetrate into the cell wall, where they disorganise and react with the cytoplasmic membrane [10]. The ionic liquid, once it has entered the cytoplasm, may interact with the negatively charged bio-polymers present, such as DNA and RNA, and complex with these, thereby altering their folding structure [11,12], and eventually cause cell death. 

Microorganisms have also a great ability for adaptating to continuously changing environmental conditions. The rate of xenobiotic conversion by microorganisms is governed by the rate of contaminant uptake and metabolism and the rate of transfer to the cell (mass transfer). Escalating microorganisms metabolic conversion capacities would not lead to higher level of transformation if mass transfer of xenobiotics to the cell is the limiting factor. This phenomenon is known as bioavailability and is controlled by a number of physicochemical processes such as sorption and desorption, diffusion, and dissolution [13].

### Previous biodegradation experiments

A number of research groups have performed biodegradability experiments on this neoteric group of chemicals. Docherty performed biodegradation tests on butyl-, hexyl- and octyl- derivatives of methylimidazolium ionic liquids with activated sewage sludge according to the OECD 301 standard [14,15]. It was found that 1-hexyl-3-methyl-imidazolium bromide and 1-methyl-3-octylimdazolium bromide were partially mineralized by the activated sludge microbial community after an extended incubation period (37 and 38 days, respectively) [15]. 

Stolte *et al*. performed ionic liquid biodegradation tests utilizing two types of microorganisms: a commercially available freeze-dried mixture of bacteria and activated sewage sludge bacteria from a municipal wastewater treatment plant [16]. As test substances they used imidazolium substituted with an alkyl chain or a short functional group (ether, hydroxyl, nitrile and others; concentrations of 0.2 mM), with simple counterions (Cl, Br, I). For imidazolium compounds containing short sidechains (≤ C_6_), functionalized or not, no biodegradation was observed. However both imidazolium ionic liquids substituted with an octyl sidechain showed complete degradation within 24 days, which matches the results reported by Docherty *et al*. [15]. Stolte *et al*. concluded that certain level of lipophilicity is a necessary condition for ionic liquids’ cations to be biodegradable, although increasing lipophilicity contributes to increasing ecotoxicity so the effort should be made to find the balance between those two aspects [16].

The concentrations applied by Docherty *et al*. (0.278 mM of [OMIM][Cl]) and Stolte *et al*. (0.2 mM [OMIM][Cl]) are as yet too low to allow for any determination of the possible effects of a large scale release in the environment or a waste water treatment plant. The concentration added to the sewage is especially important because it has been shown that a microbial inhibitory concentration (MIC) is observed. It is therefore not realistic to state a chemical is biodegradable if the MIC is low.

### Influence and effect of sorption

In addition sorption can also influence the removal of contaminants from the environment. In biodegradation tests the sorption of the analyte plays a crucial role when interpreting the results which are based on direct measurements of the removal of the analyte. We found a significant level of sorption of ILs [17] on activated sewage, contrary to the results found by Stolte [16] and Gorman-Lewis [18]. Therefore the levels of sorption cannot be ignored and play a significant role.

The aim of this paper is to determine and compare the rates of degradation of one ionic liquid at several concentrations to estimate an upper biodegradability threshold. We applied a modified OECD 301A Die-Away test to determine the biodegradability of an imidazolium ionic liquid. Due to the high concentrations of IL and sewage sludge the effect of sorption of IL will also be investigated. It was previously stated by Stolte *et al*. that one of the stages of IL breakdown is caused by dehydrogenase [16]. The activity of the sewage sludge was monitored by measuring this enzyme’s activity. The dehydrogenase activity can be applied as a standard for the determination of sewage sludge activity [19]. 

## Results and Discussion

**Figure 1:**
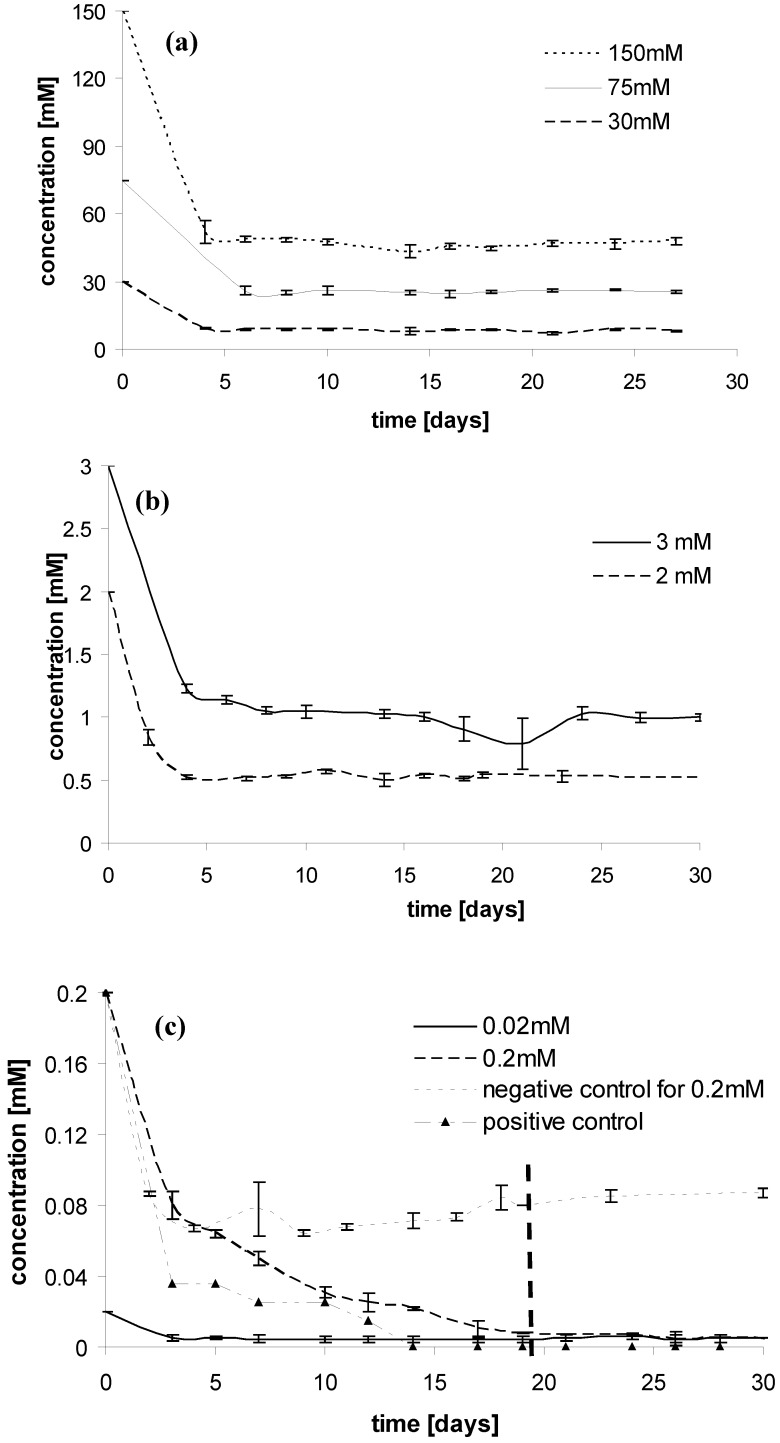
Concentration of [OMIM][Cl] in the biodegradation experiments shown in (a) with initial concentration of 150, 75, and 30 mM; (b) with initial concentration of 3, and 2 mM; (c) with initial concentration of 0.2 and 0.02 mM. The dashed line indicates the time period at which complete biodegradation has occurred. The positive control of 0.2 mM sodium benzoate is shown as a comparison. An exemplary negative control is indicated in (c).

The results, as shown in Figure 1(c), indicate that 0.2 mM of [OMIM][Cl] is completely biodegradable after ~20 days. Concentrations higher than 0.2 mM did not show any biodegradation, as displayed in Figure 1(a), Figure 1(b). In all cases we observed a decrease in the [OMIM][Cl] concentration, which reached an equilibrium after a maximum of five days. This concentration decrease was attributed to the sorption of the ionic liquid onto the sewage sludge flocs. The negative control experiments were conducted for all concentrations, and the decrease of the concentrations were found to be similar to corresponding test samples for high concentrations of IL (>0.2 mM). This would indicate the decrease in the concentration of the IL is solely due to sorption. 

**Figure 2:**
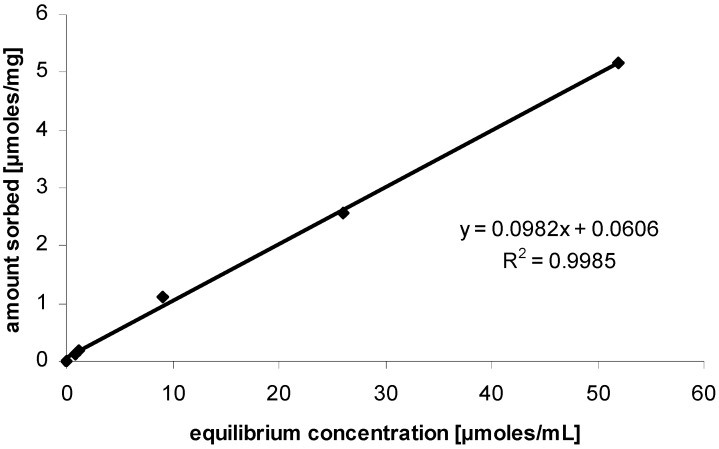
Sorption isotherm of [OMIM][Cl] on sewage sludge flocs. The data points were determined from the equilibrium concentrations of the biodegradation experiments.

In fact the equilibrium concentrations in the solution can be used to determine the amount sorbed on the sewage flocs. From this, the sorption coefficient was determined, if it is assumed that sorption was linear, as shown in Figure 2. The sorption coefficient for [OMIM][Cl] on sewage sludge flocs was determined to be 98.2 L kg^-1^. The high sorption coefficient can be attributed to the van der Waals interaction with high organic content of the sewage floc, and the alkyl chain of the imidazolium derivative. This is in agreement with previous research which clearly state that the dominant mechanism of interaction of ILs with solid matter is dependant on the lipophilicity of the IL and organic content of the solid phase [20,21].

**Figure 3:**
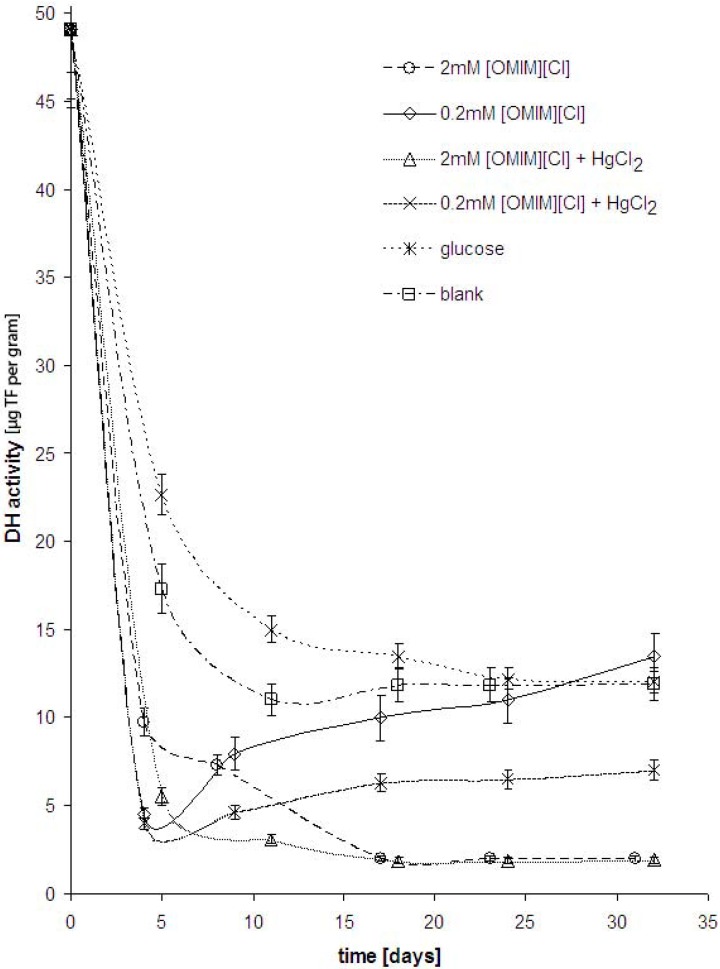
Dehydrogenase activity in six experiments, including blank, positive control with 2 mM glucose shown as µg of TF per g dry mass of flocs.

The dehydrogenase activity of 0.2 mM of [OMIM][Cl] after 25 days was comparable to that of glucose, indicating that the metabolism of the cell adapted to allow for the degradation of the ILs. When investigating the dehydrogenase activity, it was found that concentrations of 2 mM and higher of [OMIM][Cl] inhibited the enzyme activity to comparable levels as that of HgCl_2_, as shown in Figure 3. This would lead us to assume that [OMIM][Cl] at high concentrations can inhibit cell activity. Due to the structural similarity between surfactants and [OMIM][Cl] it can be thought that the mode of action is similar as to cationic surfactants such as quaternary ammonium compounds (QACs). In fact the MIC of [OMIM][Cl] for a range of yeasts and fungi was found to be between 0.5-2 mM [22]. The inhibition of the dehydrogenase activity in these experiments might be higher due to the presence of calcium. The presence of calcium can increase toxicity of some substances since Ca is known to increase membrane permeability [23].

According to the literature QACs are membrane-active agents. A target site is mainly the cytoplasmic membrane for bacteria and the plasma membrane for yeast [10]. The mode of action of surfactants includes their interaction with phospholipids components in the membrane followed by loss of selective permeability of the membrane.

## Experimental

### Modified OECD 301A DOC Die-Away Test

The ionic liquid subjected to the test was 1-methyl-3-octylmidazolium chloride [OMIM][Cl] kindly provided by the Centre for Environmental Research and Sustainable Technology, University of Bremen, Germany. The sewage sludge was taken from the aeration chamber of the ‘Gdańsk-Wschód’ municipal sewage treatment plant. The biodegradation experiment conditions were based on the OECD 301 A procedure ‘Ready Biodegradability – DOC Die-Away’ [14]. The method of degradation detection was by direct measurement of the substrate concentration by HPLC-UV. Half a litre of sewage sludge was suspended in approximately the same amount of demineralised water and mineral medium was added. Mineral medium was composed of 8.5 mg L^-1^ KH_2_PO_4_, 21.75 mg L^-1^ K_2_HPO_4_, 22.13 mg L^-1^ Na_2_HPO_4_·2H_2_O, 1.7 mg L^-1^ NH_4_Cl, 27.5 mg L^-1^ CaCl_2_, 22.5 mg L^-1^, MgSO_4_·7H_2_O and 0.25 mg L^-1^ FeCl_3_ dissolved in water. Appropriate amounts of ionic liquid stock solution were added to yield the concentration within the range of 0.02 mM to 150 mM. Flasks with test substances were accompanied by blank samples (inoculum and mineral medium without test substance) and negative controls (inoculum, mineral medium and given amount of [OMIM][Cl] poisoned with HgCl_2 _in concentration 0.4 g L^-1^) to exclude biodegradation. A positive control using 0.2 mM sodium benzoate (POCH Gliwice, Poland) was measured. All samples were continuously aerated, losses due to evaporation were adjusted on the basis of mass and analytical sample was withdrawn at specific time intervals. All experiments were conducted at least in duplicate.

### Determination of dehydrogenase activity by TTC

Dehydrogenase activity was analysed using the triphenyltetrazolium chloride (TTC) (Merck KGaA, Germany) reduction method according to the Polish Norm PN-82 C-04616.08. A triphenylformazan (TF; Fluka, Poland) standard curve was prepared by measuring the light absorbance of five TF solutions in butanol (Chempur, Poland). Sewage (50 mL) was centrifuged, the supernatant was discarded, the pellet was resuspended in buffered water and homogenised. Five mL of homogenate was added to Tris-HCl buffer, buffered water and sodium sulfite to remove oxygen. Then TTC was added and sample was incubated at 37 °C for 30 minutes. After this time the reaction was stopped by adding concentrated sulphuric acid (0.1 mL). Then butanol (10 mL) was added and the TF formed was extracted. The sample was incubated at 90 °C for 5 minutes. Five mL of organic phase was withdrawn and centrifuged. The light absorbance of clear solution was measured at 490 nm and dehydrogenase activity was calculated using the standard curve as the amount of TF per dry mass of sewage. Each extraction was conducted in duplicate. Each spectrophotometric measurement was conducted in triplicate.

### HPLC analysis

Analytical samples were centrifuged and a supernatant was taken for the HPLC/UV analysis. For ILs cation separation C_6_-Phenol (Phenomenex) 150 × 4.6 mm column was used in conjunction with detection by UV adsorption at a wavelength of 218 nm. As a mobile phase 27% acetonitrile/water + 0.1% (v/v) trifluoroacetic acid at the flow rate of 0.8 mL min^-1^ was applied. For preparation of HPLC mobile phase HPLC – grade acetonitrile form Lab – Scan (Dublin, Ireland) and spectrophotometric – grade trifluoroacetic acid (Sigma-Aldrich, Germany). Sodium benzoate was analyzed by HPLC/UV. The column used was a C_8_ Waters (XTerra) 250 × 4.6 mm with gradient elution (acetonitrile and water/acetic acid). The concentration of sodium benzoate was measured at 254 nm.

## Conclusions

In a series of biodegradation tests using various concentrations of [OMIM][Cl] it was found that the upper threshold limit for primary biodegradation was 0.2 mM using a modified OECD 301A Die-Away test. The higher concentrations caused inhibition of cell function, as was observed by a decreased dehydrogenase activity. At concentrations higher than 0.2 mM the IL was observed only to sorb onto the sewage sludge flocs. 

We have shown that the decrease in IL concentration at high concentrations of sewage and IL is mainly due to sorption on the sewage sludge flocs. Therefore care should be taken when interpreting biodegradability results of this kind of system. The sorption of ILs onto the sewage sludge flocs can be beneficial to the biodegradability as it lowers the bioavailable concentration of [OMIM][Cl] below the EC_50_ or MIC. Huang *et al*. [24] stated that the partitioning of organic compounds is to a great extend governed by the similarity (or the lack of similarity) between this organic compound and the medium into which the partitioning takes place. This suggests that compounds containing aliphatic entities will more favourably partition into aliphatic-rich matrices (like biological membranes).

The low upper threshold does conceivably limit the possible biodegradation of a concentrated release in a sewage treatment plant. Care should therefore be taken to monitor the concentrations of ILs in the wastewater streams. In addition research should be performed to increase this threshold limit, as well as determine the metabolites of the biodegradation process.
